# Prehospital Postintubation Hypotension and Survival in Severe Traumatic Brain Injury

**DOI:** 10.1001/jamanetworkopen.2025.44057

**Published:** 2025-11-20

**Authors:** James Price, Kate Lachowycz, Rob Major, Sarah McLachlan, Chris Keeliher, Benjamin Finbow, Lyle Moncur, Liam Sagi, Matt Targett, Alistair Steel, Peter B. Sherren, Ed B. G. Barnard

**Affiliations:** 1 Department of Research, Audit, Innovation, and Development, East Anglian Air Ambulance, Norwich, UK; 2 Emergency and Urgent Care Research in Cambridge, PACE Section, Department of Medicine, Cambridge University, Cambridge, UK; 3 Centre of Excellence for Equity in Uniformed Public Services, Anglia Ruskin University, Chelmsford, UK; 4 Essex & Herts Air Ambulance Trust, Earls Colne, UK; 5 Royal Infirmary of Edinburgh, NHS Lothian, Edinburgh, Scotland; 6 East of England Trauma Network, East of England, UK; 7 Magpas Air Ambulance, Huntingdon, UK; 8 Department of Critical Care Medicine, Guy’s and St Thomas’ National Health Service Foundation Trust, London, UK; 9 Academic Department of Military Emergency Medicine, Royal Centre for Defence Medicine (Research & Clinical Innovation), Birmingham, UK

## Abstract

**Importance:**

Preventing systemic disturbances, such as hypotension and hypoxia, is key to reducing the impact of secondary neuronal injury after traumatic brain injury (TBI). Postintubation hypotension is prevalent and may be associated with worse outcomes in patients with trauma undergoing emergency anesthesia.

**Objective:**

To investigate the association between postintubation hypotension and 30-day mortality in patients with severe TBI undergoing prehospital rapid sequence induction.

**Design, Setting, and Participants:**

This multicenter, retrospective, observational cohort study was performed between January 1, 2015, and December 31, 2022, in the East of England Trauma Network, including 3 helicopter emergency medical services (East Anglian Air Ambulance, Essex & Herts Air Ambulance, and Magpas Air Ambulance). A consecutive sample of patients (aged ≥16 years) with trauma and severe TBI who received prehospital rapid sequence induction by helicopter emergency medical services and were transported to a hospital within the East of England Trauma Network were eligible for inclusion. Severe TBI was defined as a Head Abbreviated Injury Scale score of 3 or higher. Data analysis was performed from March to May 2025.

**Exposure:**

Postintubation hypotension defined as a new systolic blood pressure less than 90 mmHg and induction of anesthesia at 10 minutes or less.

**Main Outcomes and Measures:**

The primary outcome was 30-day mortality.

**Results:**

A total of 555 patients (median [IQR] age, 48 [29-66] years; 408 [73.5%] male) were included in the final analysis; 548 (98.7%) had a blunt mechanism of injury. Within the first 10 minutes of anesthesia, 106 patients (19.1%) had postintubation hypotension, and 169 (30.5%) died within 30 days of injury (46 of 106 [43.4%] in the hypotension group and 123 of 449 [27.4%] in the nonhypotension group). After adjustment for confounders (eg, age and Glasgow Coma Scale score), postintubation hypotension was associated with increased 30-day mortality for patients with polytrauma and severe TBI (adjusted odds ratio [AOR], 1.70; 95% CI, 1.01-2.86; *P* = .04). For patients with isolated severe TBI who had postintubation hypotension, the odds of death adjusted for confounders (eg, age, Glasgow Coma Scale score, and Injury Severity Score) were significantly higher than for patients without (AOR, 13.55; 95% CI, 3.65-61.66; *P* < .001).

**Conclusions and Relevance:**

In this cohort study of patients with severe TBI who received prehospital rapid sequence induction, postintubation hypotension was associated with increased 30-day mortality. This association was strongest for patients with isolated TBI. These findings suggest the need for randomized prehospital interventional studies to reduce the incidence of postintubation hypotension in traumatic brain injury.

## Key Points

**Question:**

Is postintubation hypotension associated with increased 30-day mortality in patients with severe traumatic brain injury undergoing prehospital rapid sequence induction?

**Findings:**

In this cohort study of 555 patients with severe traumatic brain injury, postintubation hypotension was associated with increased mortality for patients with polytrauma and statistically significantly higher for patients with isolated traumatic brain injury.

**Meaning:**

These findings suggest the need for randomized interventional studies to reduce the incidence of postintubation hypotension in traumatic brain injury.

## Teaser

This cohort study investigates whether postintubation hypotension is associated with 30-day mortality among patients with severe traumatic brain injury undergoing prehospital rapid sequence induction.

## Introduction

Traumatic brain injury (TBI) is a leading cause of injury-related mortality and morbidity, affecting more than 50 million people worldwide each year.^1^ In England and Wales, more than 80% of patients with severe TBI are admitted to a specialist neurosurgical center and 1 in 5 is transported by helicopter emergency medical services (HEMS).^2^ After the primary injury, secondary insults from hypotension, hypoxia, and hypocapnia are prevalent in the prehospital phase of care.^3^ Suboptimal prehospital management could result in detrimental effects throughout the disease course.^1^ Early proactive management of secondary brain injury is key to improving outcomes.

Prehospital advanced airway management with rapid sequence induction (RSI) of anesthesia is necessary for a substantial proportion of severely injured patients with trauma,^4^ performed in approximately 2000 cases each year by 22 HEMS in the UK.^5^ Medical management of TBI, including the delivery of prehospital RSI, emphasizes meticulous attention to maintaining cerebral perfusion pressure by preventing and treating hypotension.^6,7^ Hypotension often occurs prior to the arrival of emergency medical service (EMS) personnel due to coexisting traumatic injury.^8^ Even brief episodes of hypotension in the early prehospital phase of resuscitation are associated with significantly increased mortality.^3,9^ Advanced airway management by HEMS is controversial, and few randomized clinical trials exist evaluating the impact of prehospital RSI in patients with severe TBI.^10^ Poorly conducted prehospital interventions are associated with increased mortality.^11^

Postintubation hypotension is prevalent, affecting more than 1 in 5 patients receiving prehospital emergency anesthesia,^12^ and may be associated with worse outcomes.^13^ The group of patients most at risk for the impact of hypotension is likely to be patients with severe TBI. The aim of this study was to investigate the association between postintubation hypotension and 30-day mortality in patients with severe TBI undergoing prehospital RSI.

## Methods

### Design

This cohort study was a post hoc secondary analysis of The Determinants of Altered Physiology in Trauma Patients Following Prehospital Emergency Anaesthesia Study,^12,21^ which has ethical approval from the Anglia Ruskin University Research Ethics Panel. This secondary analysis was approved by the UK Trauma Audit and Research Network and was registered locally and approved by each participating organization. The Strengthening the Reporting of Observational Studies in Epidemiology (STROBE) reporting guideline was followed.^20^

### Setting

This retrospective, multicenter, observational cohort study was performed in the East of England Trauma Network (EOETN) in the UK from January 1, 2015, and December 31, 2022. The East of England is large geographic region serving a population of more than 6 million people over 20 000 km^2^. Three HEMS operate within the EOETN, an inclusive trauma system with a single major trauma center (MTC) at Addenbrooke’s Hospital, Cambridge, UK.^14^ HEMS provide prehospital critical care, including prehospital RSI, on behalf of East of England Ambulance Service National Health Service Trust (EEAST), the statutory ambulance service for the region. These services are dispatched by a critical care paramedic within the EEAST emergency operations center by rotary wing or rapid response vehicle, depending on patient location, weather constraints, and time of day. Prehospital RSI is delivered by a physician and critical care paramedic team according to a shared standard operating procedure and has been previously described.^15^ This procedure includes a standardized drug regimen (ketamine, 1-2 mg/kg; rocuronium, 1 mg/kg; fentanyl, 0-3 mcg/kg).^16^

### Inclusion and Exclusion Criteria

Patients 16 years or older with severe TBI who received prehospital RSI by HEMS and were transported to a hospital within the EOETN were eligible for inclusion. Severe TBI was defined as a head Abbreviated Injury Scale (AIS) score of 3 or higher.

To evaluate for the association of postintubation hypotension independent of preexisting or preintubation hypotensive insults, patients were excluded if hypotension occurred prior to induction of anesthesia, defined as a systolic blood pressure (SBP) at the closest time preceding intubation of less than 90 mm Hg. Patients were also excluded if SBP was not available before and/or after intubation and for patients with no available outcome data (ie, transported outside the EOETN or not included in the Trauma Audit and Research Network [TARN] registry).

### Data Collection

All 3 HEMS (East Anglian Air Ambulance [EAAA], Essex & Herts Air Ambulance [EHAAT], and Magpas Air Ambulance [Magpas]) use HEMSbase (MedicOne Systems Ltd) electronic medical record software. Anonymized data were extracted from HEMSbase and collated into a password-protected data sheet (Microsoft Excel for Mac, version 16.45). The following data items were retrieved: demographics (age in years and sex), trauma type (blunt or penetrating), Glasgow Coma Scale (GCS) score, mechanism of injury, indication for prehospital RSI, fluid and vasopressor administration, and destination hospital coded as MTC or non-MTC. Data on race and ethnicity are not collected routinely and therefore are not included.

Physiologic data are routinely captured from time-calibrated patient monitors (EAAA: X Series, ZOLL Medical Corp; EHAAT and Magpas: Tempus Pro, Philips Electronics UK Ltd) and uploaded automatically to HEMSbase at 2-minute (EAAA and EHAAT) or 3-minute (Magpas) intervals. Data were manually reviewed and verified by the authors for quality.^12^ Preintubation SBP and diastolic blood pressure (DBP) were captured at the closest time point preceding the recorded RSI time. Postintubation SBP and DBP readings were captured at time points closest to 2, 4, 6, 8, and 10 minutes after intubation, with lowest postintubation SBP defined as the minimum systolic value observed across these intervals.

Outcome data were derived from the EOETN using the TARN registry. Patients are eligible for entry to the TARN registry if they sustain injuries that necessitate hospital admission and result in any of the following outcomes: 3 or more days in the hospital, admission to intensive or high-dependency care, interhospital transfer, or death from injury. Those who died at the incident scene and were not transported to the hospital are not eligible.^2^ The following data items were retrieved: Injury Severity Score (ISS), Abbreviated Injury Scale (AIS) score for each body region (head, face, chest, abdomen, spine, pelvis, limbs, and other), and 30-day survival outcome of alive or dead at 30 days. All data were anonymized before analysis and stored within a secure data environment.

### Outcome Measures

The primary outcome was the association between postintubation hypotension (defined as a new SBP <90 mm Hg at 10 minutes or less of induction)^12,17^ and 30-day mortality for patients with severe TBI (defined as head AIS score ≥3) (group 1).^18^

Secondary outcomes were the association between postintubation hypotension (defined as a new SBP <90 mm Hg at 10 minutes or less of induction)^12,17^ and 30-day mortality for patients with isolated TBI (defined as head AIS score ≥3 and all other body region AIS scores <3) (group 2).^18^

### Statistical Analysis

Data manipulation and statistical analyses were performed using the R statistical programming language, version 4.5.0 (R Foundation for Statistical Computing). Characteristics of the sample were reported as number (percentage) for categorical variables and mean (SD) or median (IQR) for continuous variables dependent on distribution of the data.

The risk-adjusted association between lowest postintubation SBP and mortality was calculated using multivariable logistic regression. Key variables were considered (ie, sex, age, GCS score, ISS, head AIS score, other region AIS score, and destination MTC vs non-MTC) based on their clinical plausibility as potential confounders. Variables were retained in the final model if they demonstrated a statistically significant association with the outcome and altered the estimated association between postintubation hypotension and the outcome by 10% or more. Model assumptions were evaluated, checking for linear associations in the logit of the outcomes, unduly influential values, and multicollinearity. All pairwise interactions among covariates were evaluated within the logistic regression model to identify potential effect modification, with a 2-sided α = .05 used without correction for multiplicity due to the exploratory nature of the analysis. Statistically significant interactions were then interrogated using stratified models and subgroup analyses. Results are presented as adjusted odds ratios (AORs) with 95% CIs. Statistical significance was predefined as a 2-sided *P* < .05. No adjustment for multiple testing was made, consistent with the exploratory design. To examine the impact of different cut-point thresholds of lowest postintubation SBP, we calculated AORs of death for values between 60 and 150 mm Hg divided into increments of 5 mm Hg using low (below the increment postintubation SBP value) vs not low (above the increment postintubation SBP value).^19^ Data analysis was performed from March to May 2025.

## Results

**Figure 1.**
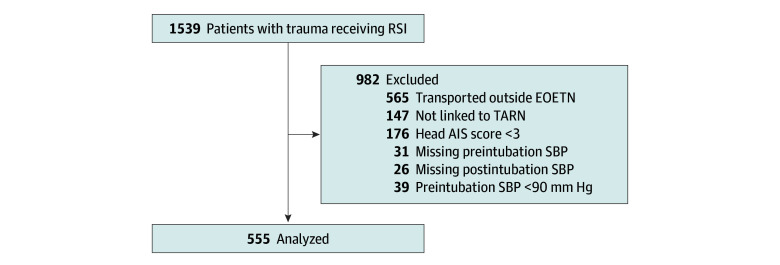
Study Flow of Patients With Trauma Attended by Helicopter Emergency Medical Services Who Received Prehospital Rapid Sequence Induction (RSI) in the East of England Trauma Network (EOETN), 2015-2022 AIS indicates Abbreviated Injury Scale; SBP, systolic blood pressure; TARN, Trauma Audit and Research Network.

During the study period, 1539 patients aged 16 years and older were attended by HEMS in the EOETN and underwent prehospital RSI after a trauma mechanism of injury, including 626 (40.7%) at the EAAA, 642 (41.7%) at the EHAAT, and 271 (17.6%) at Magpas. After applying predefined exclusions, 555 patients (median [IQR] age, 48 [29-66] years; 408 [73.5%] male and 147 [26.5%] female) were included in the final analysis (Figure 1). A total of 389 patients (70.1%) had a critical TBI (head AIS score of 5), and 226 (40.7%) had isolated severe TBI. The incidence of postintubation hypotension within the first 10 minutes after RSI was 19.1% (106 of 555). A total of 169 patients (30.5%) died within 30 days of injury, including 46 of 106 (43.4%) in the hypotension group and 123 of 449 (27.4%) in the nonhypotension group (Table).

**Table.** Characteristics of Patients With Trauma and Severe Traumatic Brain Injury Attended by Helicopter Emergency Medical Services Who Received Prehospital RSI in the East of England Trauma Network, 2015-2022

Characteristic	No. (%) of patients		
	Postintubation hypotension		Total (N = 555)
	No (n = 449)	Yes (n = 106)	
Sex			
Male	338 (75.3)	70 (66.0)	408 (73.5)
Female	111 (24.7)	36 (34.0)	147 (26.5)
Age, median (IQR), y	48 (29-65)	49 (33-68)	48 (29-66)
Injury Severity Score, median (IQR)	30 (25-38)	38 (29-45)	33 (25-43)
Glasgow Coma Scale score, median (IQR)	7 (4-9)	5 (3-9)	6 (4-9)
Trauma type			
Blunt	444 (98.9)	104 (98.1)	548 (98.7)
Penetrating	5 (1.1)	2 (1.9)	7 (1.3)
Mechanism of injury			
Unintentional injury	161 (35.9)	36 (34.0)	197 (35.5)
Assault	14 (3.1)	3 (2.8)	17 (3.1)
Self-harm	5 (1.1)	7 (6.6)	12 (2.2)
Sport or leisure	15 (3.3)	4 (3.8)	19 (3.4)
Transport	254 (56.6)	56 (52.8)	310 (55.9)
Indication for RSI			
Reduced consciousness	165 (46.6)	45 (51.7)	210 (47.6)
Airway obstruction or compromise	83 (23.5)	20 (23.0)	103 (23.4)
Ventilatory failure	23 (6.5)	9 (10.3)	32 (7.3)
Agitated head injury	34 (9.6)	6 (6.9)	40 (9.1)
Anticipated clinical course	19 (5.4)	4 (4.6)	23 (5.2)
Other or missing	125 (27.8)	22 (20.8)	147 (26.5)
Pre-RSI fluids			
None	347 (77.3)	71 (67.0)	418 (75.3)
Fluids given	102 (22.7)	35 (33.0)	137 (24.7)
Vasopressor use			
Given	39 (8.7)	35 (33.0)	74 (13.3)
Not given	370 (82.4)	54 (50.9)	424 (76.4)
Unknown	40 (8.9)	17 (16.0)	57 (10.3)
Head AIS			
3	32 (7.1)	13 (12.3)	45 (8.1)
4	104 (23.2)	16 (15.1)	120 (21.6)
5	312 (69.5)	77 (72.6)	389 (70.1)
6	1 (0.2)	0	1 (0.2)
Other body region AIS			
<3	203 (45.2)	23 (21.7)	226 (40.7)
≥3	246 (54.8)	83 (78.3)	329 (59.3)
Destination hospital			
MTC	419 (93.3)	88 (83.0)	507 (91.4)
Non-MTC	30 (6.7)	18 (17.0)	48 (8.7)
30-d Mortality			
Alive	326 (72.6)	60 (56.6)	386 (69.6)
Dead	123 (27.4)	46 (43.4)	169 (30.5)

Abbreviations: AIS, Abbreviated Injury Scale; MTC, major trauma center; RSI, rapid sequence induction.

After adjustment for confounders, postintubation hypotension was significantly associated with increased 30-day mortality for patients with polytrauma and severe TBI (AOR, 1.70; 95% CI, 1.01-2.86; *P* = .04) (eTable 1 in Supplement 1). There was a statistically significant interaction between postintubation hypotension and ISS (*P *for interaction = .03). Further analysis revealed that the association between hypotension and mortality was more pronounced in patients with lower ISS scores, which is explored further in the subgroup analysis of patients with isolated TBI who have lower ISS scores. The overall model demonstrated a better fit when ISS was excluded, and its omission had little influence on the strength of the association between hypotension and mortality.

For patients with isolated severe TBI who had postintubation hypotension, the adjusted odds of death were significantly higher compared with patients without (AOR, 13.55; 95% CI, 3.65-61.66; *P* < .001) (eTable 2 in Supplement 1). There was no longer any significant interaction between ISS and postintubation hypotension in this subgroup analysis. There was no significant interaction among sex, destination hospital, and other body region AIS scores.

Head AIS score was significantly associated with the survival outcome. However, inclusion of this variable as a confounder increased the AORs for the association between postintubation hypotension and mortality (AOR, 44.50; 95% CI, 8.0-447.51). Further examination found that head AIS scores exhibited statistical issues, including noncollapsibility (ie, strata-specific ORs were higher than the composite estimate), an unbalanced distribution with small subgroup sizes, and a significant interaction with postintubation hypotension. eFigure 1 in Supplement 1 shows the mortality rate by head AIS score and postintubation hypotension status for the isolated TBI subgroup. In patients with a head AIS score of 5 who had postintubation hypotension, the mortality rate was 94% compared with 37% for those without postintubation hypotension; patients with a head AIS score of 3 were not included due to small subgroup (n = 13) and 0% mortality. Due to limited sample size in the group with a head AIS score of 4 (n = 52), stratified regression results were not presented because they were unstable and potentially misleading. eTable 3 in Supplement 1 presents AORs for the association between postintubation hypotension and mortality in the group with a head score of 5.

**Figure 2.**
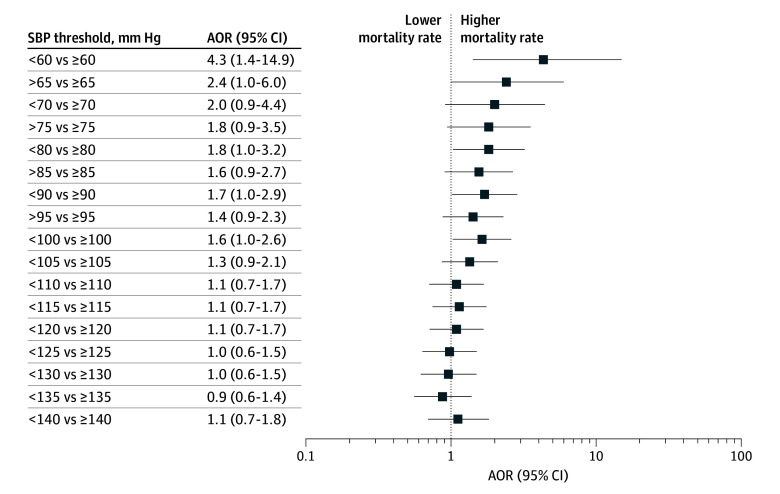
Adjusted Odds Ratios (AORs) of Death for Lowest Postintubation Systolic Blood Pressure (SBP) in Patients With Polytrauma and Severe Traumatic Brain Injury (Group 1) in the East of England Trauma Network, 2015-2022^a^ ^a^Severe traumatic brain injury was defined as Head Abbreviated Injury Scale Score ≥3.

**Figure 3.**
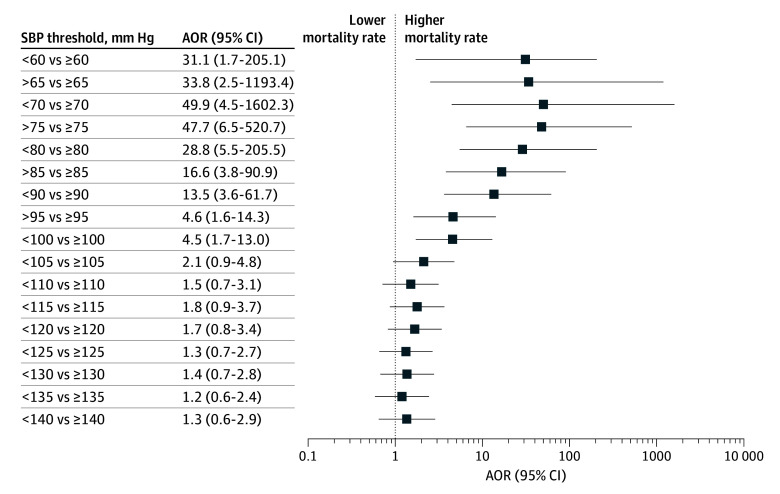
Adjusted Odds Ratios (AORs) of Death for Lowest Postintubation Systolic Blood Pressure (SBP) in Patients With Trauma and Isolated Severe Traumatic Brain Injury (Head Abbreviated Injury Scale Score ≥3 and All Other Body Region Abbreviated Injury Scale Score <3) in the East of England Trauma Network, 2015-2022 ^a^Isolated severe traumatic brain injury was defined as head Abbreviated Injury Scale score ≥3 and all other body region Abbreviated Injury Scale score <3.

Figure 2 and Figure 3 show the distribution of ORs of death by cut-point thresholds of lowest postintubation SBP in increments of 5 mm Hg from 60 to 140 mm Hg for all patients with severe TBI (Figure 2) and for the subgroup of patients with isolated TBI (Figure 3). Statistically significant differences in mortality were observed for postintubation SBP up to 100 mm Hg (Figure 2 and Figure 3). Among patients with isolated brain injury, the ORs for cut points in the lower blood pressure range (<90 mm Hg) have wide CIs, reflecting the small sample size.

## Discussion

This study found that for patients with trauma and severe TBI undergoing prehospital RSI, there was a significant increase in 30-day mortality for patients who had postintubation hypotension compared with those without. This association was strongest for patients with the most severe isolated TBI and was observed at cut points of postintubation SBP up to 100 mm Hg within the first 10 minutes of anesthesia.

Advanced airway management with RSI is required for a subset of critically injured patients with trauma.^4^ Almost 9 in 10 patients in England with severe TBI have definitive airway management before or shortly after hospital arrival.^2^ Prehospital RSI aims to optimize conditions for tracheal intubation and minimize the impact of secondary neuronal injury through prevention of systemic disturbances, such as hypotension, hypoxemia, and hypocapnia.^22,23^ Postintubation hypotension is frequently observed in patients with trauma undergoing prehospital RSI,^12^ with some work^24^ reporting an incidence of approximately two-thirds for patients with isolated TBI. This study demonstrates that nearly 1 in 5 patients with severe TBI who undergo prehospital RSI experiences postintubation hypotension. Previous work^13^ has reported an association between postintubation hypotension and mortality in patients with trauma undergoing RSI in the hospital. However, the current study demonstrates this effect in the hyperacute phase of resuscitation with higher mortality in specific subgroups of patients and particularly those with more severe isolated TBI.

The avoidance of secondary brain insult is critical to improving outcomes for patients with severe TBI,^1,6^ particularly for patients with the most severe injury in which autoregulatory mechanisms are impaired.^25^ This study supports a recent pooled meta-analysis^26^ demonstrating an association between prehospital hypotension and adverse outcomes in patients with severe TBI, with the highest mortality observed in the isolated TBI groups. Cerebral autoregulation plays a crucial role in preserving stable cerebral blood flow and brain oxygenation in response to fluctuations in arterial blood pressure.^27^ When autoregulation is intact, a reduction in SBP elicits a compensatory vasodilatory response within the cerebral vasculature to preserve cerebral perfusion. Dysautoregulation is frequently observed in patients with more severe TBI.^28^ When autoregulation is compromised, cerebral blood flow becomes pressure passive and directly dependent on systemic SBP, with hypotension precipitating cerebral ischemia.^25^ As a result, these patients are the most at risk for hypotensive injury and subsequent cerebral ischemia, which may account for the increased mortality observed in this group.

The definition and threshold for hypotension in TBI have been the subject of much debate, with literature definitions of blood pressures varying widely from 80 to 120 mm Hg.^19^ Frequently referred to as a cut point of less than 90 mm Hg, the previous iteration of the Brain Trauma Foundation guidelines highlighted the hypotension threshold as a key priority for future research.^29^ Notable work by Spaite et al^19^ describes the association between SBP and the adjusted log odds of death as linear rather than a specific cut point. In this study, a similar trajectory model is observed, suggesting that the most recent increased hypotension threshold of 110 mm Hg^25^ may be most appropriate for patients with more serious head injuries and that higher SBPs may be beneficial during the prehospital and resuscitation phase of care.^6^

Traditional induction agents for prehospital RSI have increasingly been replaced by drugs offering greater physiologic stability, notably ketamine, in critically ill patients.^22,30^ In patients who are neither injured nor hemodynamically compromised, ketamine’s vasodilatory and direct negative inotropic effects are effectively mitigated by its centrally mediated sympathomimetic action.^31^ However, this study includes patients with severe TBI, likely accompanied by a degree of brain injury–induced autonomic dysfunction; thus, the capacity to elicit a sufficient sympathomimetic response is impaired.^32^ As a result, ketamine’s negative inotropic influence becomes the predominant effect. This effect, in addition to relative hypovolemia in trauma and the introduction of positive pressure ventilation, impedes preload and culminates in the profound hypotension observed in this study.

Recognizing this pharmacopathological association, emergency anesthesia guidelines recommend the administration of intravenous fluid to increase the intravascular volume before induction of anesthesia.^30^ The Excellence in Prehospital Injury Care study conducted in Arizona introduced statewide prehospital TBI guidelines with a focus on avoidance and treatment of hypotension using isotonic fluids.^33^ The results demonstrated significant improvement in postimplementation adjusted survival for severe TBI, particularly for patients who received positive pressure ventilation and/or prehospital intubation. Comparable improvements have also been observed with the administration of prehospital plasma transfusion and preinduction hypertonic saline as potential therapies to mitigate postintubation hypotension.^24,34^ However, it is important to acknowledge that this study identifies an association between postintubation hypotension and increased mortality rather than establishing a causal relationship. Although aggressive prevention and proactive management of postintubation hypotension are recommended,^35^ these interventions may not necessarily translate into improved clinical outcomes. It is possible that postintubation hypotension serves as a surrogate marker for the most critically brain injured patients, in whom high mortality may be largely irreversible. Understanding this association necessitates further interventional research aimed at mitigating hemodynamic embarrassment after prehospital RSI.

### Limitations

This study has some limitations. The study included a large, heterogeneous cohort of patients and clinicians operating within 3 HEMS organizations in a single trauma network in the UK. Although this limits external generalizability, there are advantages to this approach. All participating HEMS operate under the same prehospital RSI standard operating procedure, serving a similar patient population and referring into a consistent system of onward hospital care. This reduces variability in clinical practice and controls for potential confounders related to the induction drug or dose regimen, monitoring practices, and in-hospital management that may influence outcomes. External validation in other health systems would be valuable to confirm the broader applicability of our findings.

The available physiologic data were limited to those captured by the participating HEMS organizations, with no data recorded before the arrival of HEMS available for inclusion. Consequently, it was not possible to adjust for these variables before HEMS arrival. To independently assess the impact of postintubation hypotension, patients with documented preinduction hypotension were excluded from the analysis. It is likely that some patients who experienced hypotensive insults before HEMS arrival were inadvertently included, which may have led to an overestimation of the effect.

Data were manually reviewed and verified by the authors for quality.^12^ However, 26 patients were excluded due to missing postintubation SBP readings. Although in some cases this may have resulted from equipment or technical failure, it is possible that these patients experienced sudden cardiovascular collapse without preceding changes in SBP. This possibility represents a potential source of bias and may have led to an underestimation of the true incidence of postintubation cardiovascular collapse. Additionally, some subgroup analyses, such as those stratified by head AIS scores, involved relatively small numbers of patients. This issue contributed to wide CIs, which should be considered when interpreting the precision of these estimates.

## Conclusions

In this cohort study of patients with trauma and severe TBI who received prehospital RSI, postintubation hypotension was associated with increased 30-day mortality. This association was strongest for patients with isolated TBI (head AIS score ≥3 and all other body region AIS <3). These findings suggest the need for randomized prehospital interventional studies to reduce the incidence of postintubation hypotension in TBI.

## Notes

Supplement 1. **eTable 1.** Group 1: Adjusted odds ratios of death for adult polytrauma patients with severe traumatic brain injury (Head AIS ≥3) who had postintubation hypotension, in the East of England Trauma Network; 2015-2022, *n*=555 **eTable 2.** Group 2: Adjusted odds ratios of death for adult trauma patients with isolated severe traumatic brain injury (Head AIS ≥3 and all other body region AIS <3) who had postintubation hypotension, in the East of England Trauma Network; 2015-2022, *n*=225 **eTable 3. **Group 2 for Head AIS 5: Adjusted odds ratios of death for adult trauma patients with isolated critical traumatic brain injury (Head AIS =5 and all other body region AIS <3) who had postintubation hypotension, in the East of England Trauma Network; 2015-2022, *n*=159 **eFigure.** Group 2: Mortality rate for adult trauma patients with isolated severe traumatic brain injury (Head AIS ≥3 and all other body region AIS <3) dichotomised into Head AIS =4 and Head AIS =5, in the East of England Trauma Network; 2015-2022

Supplement 2.
Data Sharing Statement
