# Supplementary material for: Prehospital Postintubation Hypotension and Survival in Severe Traumatic Brain Injury
Source: JAMA Netw Open. 2025 Nov 20;8(11):e2544057. doi: 10.1001/jamanetworkopen.2025.44057 (PMC12635874; doi:10.1001/jamanetworkopen.2025.44057)
Supplement: Supplement 1. — eTable 1. Group 1: Adjusted odds ratios of death for adult polytrauma patients with severe traumatic brain injury (Head AIS ≥3) who had postintubation hypotension, in the East of England Trauma Network; 2015-2022, n=555 eTable 2. Group 2: Adjusted odds ratios of death for adult trauma patients with isolated severe traumatic brain injury (Head AIS ≥3 and all other body region AIS <3) who had postintubation hypotension, in the East of England Trauma Network; 2015-2022, n=225 eTable 3. Group 2 for Head AIS 5: Adjusted odds ratios of death for adult trauma patients with isolated critical traumatic brain injury (Head AIS =5 and all other body region AIS <3) who had postintubation hypotension, in the East of England Trauma Network; 2015-2022, n=159 eFigure. Group 2: Mortality rate for adult trauma patients with isolated severe traumatic brain injury (Head AIS ≥3 and all other body region AIS <3) dichotomised into Head AIS =4 and Head AIS =5, in the East of England Trauma Network; 2015-2022 [file jamanetwopen-e2544057-s001.pdf]

Supplemental Online Content

Price J, Lachowycz K, Major R, et al. Prehospital postintubation hypotension and survival in severe traumatic brain injury. *JAMA Netw Open*. 2025;8(11):e2544057. doi:10.1001/jamanetworkopen.2025.44057

eTable 1. Group 1: Adjusted odds ratios of death for adult polytrauma patients with severe traumatic brain injury (Head AIS ≥3) who had postintubation hypotension, in the East of England Trauma Network; 2015-2022, *n*=555

eTable 2. Group 2: Adjusted odds ratios of death for adult trauma patients with isolated severe traumatic brain injury (Head AIS ≥3 and all other body region AIS <3) who had postintubation hypotension, in the East of England Trauma Network; 2015-2022, *n*=225

eTable 3. Group 2 for Head AIS 5: Adjusted odds ratios of death for adult trauma patients with isolated critical traumatic brain injury (Head AIS =5 and all other body region AIS <3) who had postintubation hypotension, in the East of England Trauma Network; 2015-2022, *n*=159

eFigure. Group 2: Mortality rate for adult trauma patients with isolated severe traumatic brain injury (Head AIS ≥3 and all other body region AIS <3) dichotomised into Head AIS =4 and Head AIS =5, in the East of England Trauma Network; 2015-2022

This supplemental material has been provided by the authors to give readers additional information about their work.

© 2025 Price J et al. *JAMA Network Open.*

eTable 1: Group 1: Adjusted odds ratios of death for adult polytrauma patients with severe traumatic brain injury (Head AIS ≥3) who had postintubation hypotension, in the East of England Trauma Network; 2015-2022, *n*=555.

**Variable**	**Adjusted Odds Ratio (95%CI)**	***p*-value**
Postintubation hypotension (new SBP <90mmHg ≤10 min of induction)	1.70 (1.01-2.86)	0.04
Age	1.04 (1.03-1.06)	<0.001
GCS score	0.71 (0.64-0.76)	0.04

McFadden's Psuedo R^2 = 0.24
AIS Abbreviated Injury Scale (score), CI Confidence Interval, GCS Glasgow Coma Scale, ISS Injury Severity Score, SBP Systolic Blood Pressure

© 2025 Price J et al. *JAMA Network Open.*

eTable 2: Group 2: Adjusted odds ratios of death for adult trauma patients with isolated severe traumatic brain injury (Head AIS ≥3 and all other body region AIS <3) who had postintubation hypotension, in the East of England Trauma Network; 2015-2022, *n*=225.

Variable	Adjusted Odds Ratio (95%CI)	p-value
Postintubation hypotension<br>(new SBP <90mmHg ≤10 min of induction)	13.55 (3.65 - 61.66)	<0.001
Age	1.04 (1.02-1.07)	<0.001
GCS score	0.66 (0.55-0.76)	<0.001
ISS	1.21 (1.11-1.35)	<0.001

McFadden's Psuedo R^2 = 0.37
AIS Abbreviated Injury Scale (score), CI Confidence Interval, GCS Glasgow Coma Scale, ISS Injury Severity Score, SBP Systolic Blood Pressure

© 2025 Price J et al. *JAMA Network Open.*

eTable 3 : Group 2 stratified by Head AIS 5: Adjusted odds ratios of death for adult trauma patients with isolated critical traumatic brain injury (Head AIS =5 and all other body region AIS <3) who had postintubation hypotension, in the East of England Trauma Network; 2015-2022, *n*=159

**Variable**	**Adjusted Odds Ratio (95%CI)**	**p-value**
Postintubation hypotension (new SBP <90mmHg ≤10 min of induction)	**108.39 (10.56 – 3477.07)**	<0.001
Age	**1.05 (1.02 – 1.07)**	<0.001
GCS score	**0.65 (0.53 – 0.77)**	<0.001

McFadden's Psuedo R^2 = 0.37
AIS Abbreviated Injury Scale (score), CI Confidence Interval, GCS Glasgow Coma Scale, SBP Systolic Blood Pressure

© 2025 Price J et al. *JAMA Network Open.*

eFigure 1: Group 2: Mortality rate for adult trauma patients with isolated severe traumatic brain injury (Head AIS ≥3 and all other body region AIS <3) dichotomised into Head AIS =4 and Head AIS =5, in the East of England Trauma Network; 2015-2022.

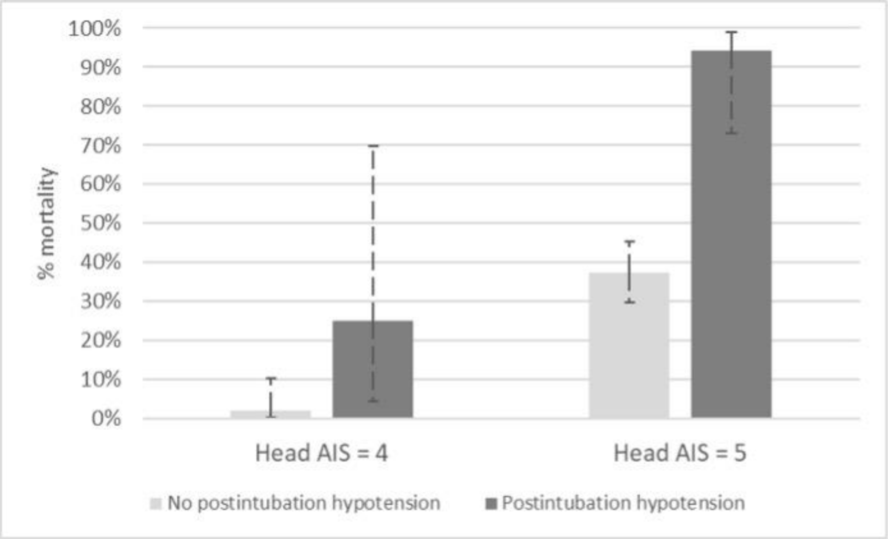

AIS Abbreviated Injury Scale (score)
|----| 95% confidence intervals using Wilson Score method.

© 2025 Price J et al. *JAMA Network Open.*
