# Supplementary material for: Prehospital Postintubation Hypotension and Survival in Severe Traumatic Brain Injury
Source: JAMA Netw Open. 2025 Nov 20;8(11):e2544057. doi: 10.1001/jamanetworkopen.2025.44057 (PMC12635874; doi:10.1001/jamanetworkopen.2025.44057)
Supplement: Supplement 2. — Data Sharing Statement [file jamanetwopen-e2544057-s002.pdf]

Data Sharing Statement

Price. Prehospital Postintubation Hypotension and Survival in Severe Traumatic Brain Injury. *JAMA Netw Open*. Published November 20, 2025. doi:10.1001/jamanetworkopen.2025.44057

Data
Data available: No

Additional Information
Explanation for why data not available: The datasets used and/or analyzed during the current study are available on reasonable request.
